# Lipotoxicity-related sarcopenia: a review

**DOI:** 10.25122/jml-2022-0157

**Published:** 2022-11

**Authors:** Rashid Abdulhameed Aldahhan, Kamaluddin Hasan Motawei, Mohammed Taha Al-Hariri

**Affiliations:** 1 Department of Anatomy, College of Medicine, Imam Abdulrahman Bin Faisal University, Dammam, Saudi Arabia; 2 Department of Physiology, College of Medicine, Imam Abdulrahman Bin Faisal University, Dammam, Saudi Arabia

**Keywords:** sarcopenia, lipotoxicity, mitochondrial dysfunction, pro-inflammatory cytokines, oxidative stress

## Abstract

A body of literature supports the postulation that a persistent lipid metabolic imbalance causes lipotoxicity, “an abnormal fat storage in the peripheral organs”. Hence, lipotoxicity could somewhat explain the process of sarcopenia, an aging-related, gradual, and involuntary decline in skeletal muscle strength and mass associated with several health complications. This review focuses on the recent mechanisms underlying lipotoxicity-related sarcopenia. A vicious cycle occurs between sarcopenia and ectopic fat storage via a complex interplay of mitochondrial dysfunction, pro-inflammatory cytokine production, oxidative stress, collagen deposition, extracellular matrix remodeling, and life habits. The repercussions of lipotoxicity exacerbation of sarcopenia can include increased disability, morbidity, and mortality. This suggests that appropriate lipotoxicity management should be considered the primary target for the prevention and/or treatment of chronic musculoskeletal and other aging-related disorders. Further advanced research is needed to understand the molecular details of lipotoxicity and its consequences for sarcopenia and sarcopenia-related comorbidities.

## INTRODUCTION

Several experimental and clinical studies have shown an association between advanced age and an inevitable gradual decrease in skeletal muscle strength and mass, known as sarcopenia [1]. Sarcopenia usually begins in the fifth decade of life and has been linked to an increased incidence of falls and fractures [2], as well as a loss of functionality and independence [3], which leads to increased morbidity and/or mortality [4]. Sarcopenia is histologically characterized by a reduction in the cross-bridging components between muscle fibers, smaller and/or fewer mitochondria in muscle cells, atrophy of type II myofibers, and tissue necrosis [1]. Published evidence has also shown that adipose tissue infiltration of the skeletal muscle predicts a loss of muscle power in the elderly, even in those who maintain a healthy weight [4].

Adipose tissue is an immune endocrine organ that also serves an energy storage function [5]. Triglycerides are hydrolyzed intracellularly by lipases into free fatty acids and glycerol for transportation to extra-adipose tissues, where they are oxidized by mitochondria. If the hydrolysis process exceeds the capacity to esterify intracellular free fatty acids, the resulting net release of free fatty acids can have many adverse effects, such as cytotoxicity, ectopic storage, and susceptibility to lipotoxicity insult [6, 7]. In aging humans, despite an increase in the total percentage of visceral fat, the capacity of white adipose tissue (lipid storage) to buffer plasma non-esterified fatty acids (the end products of fasting lipolysis) diminishes due to impaired adipogenesis [2]. Obesity causes a further formation of excessive triglyceride deposits, known as steatosis or ectopic fat deposits, in several tissues, such as muscle, heart, pancreas, and liver [8, 9].

The metabolic profile of skeletal muscle fibers is either more glycolytic (essentially using glucose) in rapid-firing type II fibers or more oxidative (essentially using lipids) in slow-firing type I fibers [10, 11]. While fatty acid oxidation is relatively high in the skeletal muscle, lipid overload could also occur, eventually triggering muscle cell death through insulin resistance and other mechanisms.

## MECHANISMS UNDERLYING THE DEVELOPMENT OF LIPOTOXICITY-RELATED SARCOPENIA

**Figure 1:**
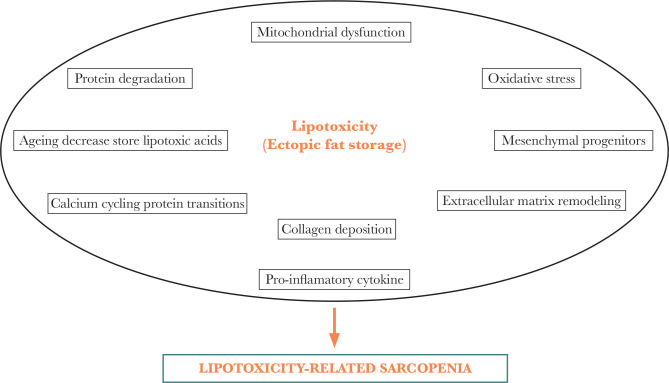
Integrated mechanisms caused by lipotoxicity in sarcopenia.

Lipotoxicity is a systemic disorder associated with metabolic and senescence diseases, such as obesity and sarcopenia. The pathogenesis of lipotoxicity-related sarcopenia takes place through a cascade of intermingled mechanisms. Lipotoxicity leads to ectopic storage of lipids in the skeletal muscles (myosteatosis) and enhances the release of adipokines, cytokines, and chemokines, eventually leading to chronic sterile inflammation of muscles and impaired function of their mitochondria. The end result is a reduced capacity to consume fatty acids, followed by oxidative stress, insulin resistance, calcium store depletion, protein degradation, and extracellular matrix changes (Figure 1).

### Myosteatosis

In lean individuals, triacylglycerol (TAG) normally represents 0.5% of the skeletal muscle volume, but this percentage can increase to 3.5% in obesity [12]. The increase in body fat mass is associated with ectopic fat deposits that occur preferentially in muscles. This is termed myosteatosis [9] and appears to act synergistically with sarcopenia, as shown in Figure 1. Myosteatosis should be regarded as a physiologically accelerated degenerative process arising due to concomitant lipotoxic stress [3, 13]. It materializes as intramyocellular lipids that accumulate due to an increased inflow of fatty acids that exceeds the oxidative capacity of skeletal muscles [14] and the intramyocellular adipocytes of the extramuscular adipose tissue as a result of stimulation of adipogenic metabolism. Some research has shown that aged persons with high muscle fat infiltration in the mid-thigh have a high incidence of mobility impairment during a 2.5-year follow-up period [15]. Similarly, fat accumulation in the middle-aged can develop into fibrosis, further impairing muscle movement and function [16].

### Chronic sterile low-grade inflammation

Adipose tissue secretes many different factors, including pro-inflammatory cytokines, extracellular matrix proteins, pro-thrombotic factors, and chemokines [17]. Macrophages also release pro-inflammatory cytokines that activate a large number of stress-signaling cascades. These cascades upregulate CD11c surface expression in adipose-resident macrophages and stimulate them to assume a pro-inflammatory secretory profile [18]. The released chemical factors exacerbate and trigger other stress-signaling cascades, causing the release of free fatty acids and, ultimately, lipotoxicity [19].

Crosstalk has recently been identified between inflamed skeletal muscle and adipose tissue, generating an age-related and harmful vicious cycle that may be the key conjoining mechanism between lipotoxicity and sarcopenia [12, 20]. Sequences of pro-inflammatory cytokine signaling and cellular stress responses are triggered by lipotoxicity, thereby depleting the preadipocyte progenitor pool. The muscles then switch to a pro-inflammatory condition similar to that of macrophages [21].

These changes are exacerbated by aging, as skeletal muscle fibers become damaged by fatty acids and inflammation while also losing their capacity to store lipotoxic acids. The further release of pro-inflammatory cytokine signals and the vicious feedback loop has a profound impact on skeletal muscle fibers and motor function and can play a significant role in sarcopenia [22].

### Oxidative stress

Many studies have shown that feeding a high-fat diet increases reactive oxygen species and causes nitric oxide imbalances, thereby altering cellular antioxidant defense systems. The result is cellular membrane disruption, decreased protein synthesis due to endoplasmic reticulum stress, and activation of muscle fiber apoptosis [23]. In elderly individuals, changes in the intramyocellular ultrastructure have been correlated with transcriptional alterations related to mitochondrial dysfunction and lipid metabolism [24]. Increased levels of reactive oxygen species in type I muscle fibers, and disturbances in cellular homeostasis predispose muscles to impairments in the function and integrity of neuromuscular junctions [25].

### Insulin resistance

**Figure 2:**
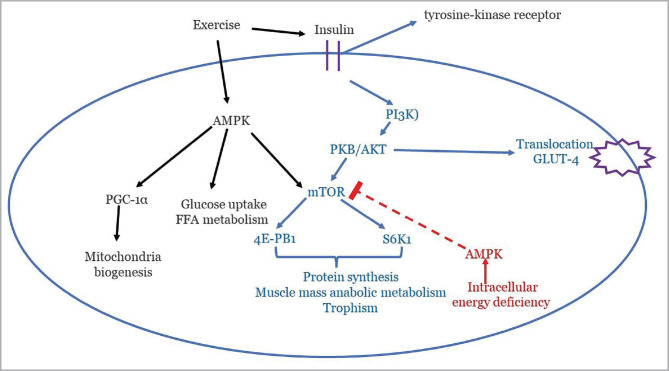
Molecular pathways of diet and exercise which are compromised during aging. PGC-1α, peroxisome proliferator receptor gamma activator 1α; FFA, free fatty acids; PI3K, phosphatidylinositol 3-kinase; PKB/AKT, protein kinase B; GLUT, glucose transporter; mTOR, mammalian target of rapamycin; 4E-PB1, 4E-binding protein 1; S6K1, ribosomal S6 kinase 1; AMPK, AMP-activated kinase.

In skeletal muscle, exercise and binding insulin with its tyrosine-kinase receptors exert several biological effects, including protein synthesis and glucose metabolism (Figure 2). Auto-phosphorylation of the receptor leads to the recruitment of insulin receptor substrate (IRS)-1, which guides downstream pathways [26]. When the phosphatidylinositol 3-kinase (PI3K) is activated, it promotes phosphorylation of protein kinase B (PKB)/AKT and allows the internalization of glucose by translocation of glucose transporter (GLUT)-4. The phosphorylation of glycogen synthase kinase 3 (GSK3) promotes glycogen synthesis. All of these mechanisms aim to store and dispose of glucose. Additionally, PKB/AKT stimulates the mammalian target of rapamycin (mTOR), ribosomal S6 kinase 1 (S6K1), and 4E-binding protein 1 (4E-PB1), which are involved in the importance of tropism, muscle mass anabolic metabolism and protein synthesis [26].

A more important signaling pathway is represented by the AMP-activated kinase (AMPK), which promotes free fatty acids and glucose metabolism as well as modulates long-term responses in mitochondria by interacting with peroxisome proliferator receptor-gamma activator 1α (PGC-1α) [27]. In the presence of intracellular energy deficiency, AMPK inhibits protein synthesis by suppressing mTOR signaling [28].

One direct effect of insulin on the muscle phenotype is the suppression of protein catabolism [29]. Insulin resistance is a senescence morbidity reciprocally associated with sarcopenia [30]. Insulin resistance inhibits β-oxidation, increases the supply of free fatty acids, and alters triglyceride transport, resulting in steatosis [31] and suppression of the growth hormone (GH)-insulin-like growth factor 1 (IGF1) axis responsible for muscle protein synthesis [32]. The hyperinsulinemia resulting from insulin resistance directly accelerates muscle degradation and decelerates protein synthesis [33], thereby leading to an increased production of myostatin that reduces muscle mass [34].

### Leptin resistance

**Figure 3:**
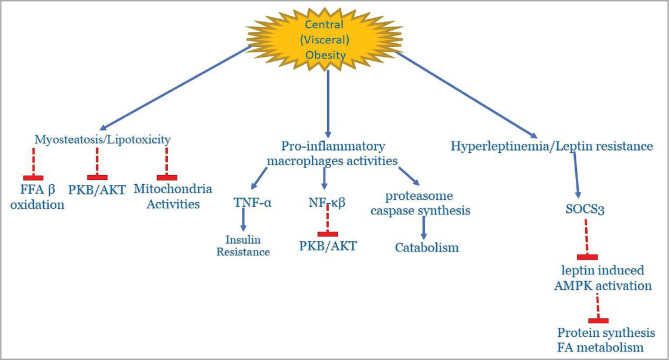
Schematic pathological pathways in central obesity contributed to the development of sarcopenia. SOCS3, suppressors of cytokine signaling 3; TNF-α, Tumor Necrosis Factor – α; PKB/AKT, Protein kinase B/AKT; FFA, free fatty acids; NF-κB, Nuclear Factor-κB; FA, fatty acids; AMPK, AMP-activated protein kinase.

Central (visceral) obesity is a well-known pathological condition where the adipose tissue represents an actively secreting organ, contributing to the release of several pro-inflammatory cytokines that enhance local and systemic inflammation (Figure 3).

Leptin, secreted by adipose tissue, acts as a pro-inflammatory hormone, especially in subjects with sarcopenic obesity rather than in those with either visceral obesity or sarcopenia alone [4]. Hyperleptinemia could be due to defective signaling at the hypothalamic neurons and leptin resistance [35].

In healthy subjects, leptin stimulates AMPK in skeletal muscles. Meanwhile, this pathway is suppressed in obese individuals, which is attributed to the increased hypothalamic expression of the obesity-related suppressors of cytokine signaling 3 (SOCS3). In the experimental model, SOSC3 inhibits leptin activation of AMPK, contributing to the impaired fatty acid metabolism in skeletal muscle [36].

In an obesogenic mimicking environment, studies showed that macrophages interfere with muscle function “by decreasing phosphorylated PKB/AKT and nuclear factor (NF)-κβ inhibiting protein (Inhibiting κβ-α [Iκβ-α])” [37].

### Extracellular matrix remodeling

The extracellular matrix (ECM) plays crucial roles in skeletal muscle development [38], biomechanics [39–41], regeneration [42, 43], motor endplate function [44, 45], and glucose metabolism [46]. Consequently, the pathophysiologies of many skeletal muscle diseases, such as different varieties of muscular dystrophy [47] and senescence-associated sarcopenia [48, 49], likely involve a remodeling of ECM components. ECM remodeling is associated with the consumption of high-fat diets, and an increased collagen content has an apparent association with insulin resistance in skeletal muscles [46]. The collagen deposition and extracellular matrix remodeling triggered by lipotoxicity cause changes in the functionality of the sarcoplasmic reticulum, resulting in impaired fiber contractility [50]. An involvement of lipotoxicity is claimed in tubulointerstitial fibrosis in the kidney due to the increased expression of connective tissue growth factors and the promotion of apoptosis [51]. However, evidence for the operation of a similar mechanism in lipotoxicity-related sarcopenia is presently lacking.

### Calcium imbalance

Lipid stress may cause transitions in calcium cycling protein isoforms, thereby influencing calcium homeostasis, calcium signaling, and muscle twitch (fiber excitation, contraction, and relaxation) [52, 53]. Sarcopenia is associated with the dysfunctional enlargement of mitochondria, which increases mitochondrial Ca^2+^ uptake and depletes calcium in the myofibers [54].

### Myofiber atrophy

A synergistic relationship exists between muscle loss and skeletal muscle fatty infiltration, suggesting that fat accumulation might accelerate the pathogenesis of sarcopenia [13]. In aged rats, a high-fat diet results in intramyocellular accumulation of fatty acids. This increase in fatty acids is correlated with impaired skeletal muscle protein synthesis [55] and may indicate that the increased accumulation of lipid metabolism byproducts, such as ceramide, has adverse effects on mitochondrial performance [56]. By contrast, the accumulation of adipocytes in skeletal muscles has negative effects on the muscle phenotype and promotes muscle atrophy, as shown in both humans and rats by Pellegrinelli and collaborators [57]. Impaired autophagy and reduced numbers of satellite cells, as occur in overweight-related sarcopenia, further contribute to muscle wasting [58].

Lipotoxicity triggers cell death in smooth muscles [59] as well as in cardiac muscles [60]. The result is smooth muscle cell proliferation, muscle remodeling, pathological alterations in vascular tone, vascular foam cell formation, and plaque destabilization. In the heart, the effects can include abnormal right ventricle geometry, increased left ventricular mass, enlarged atrial chemotaxis, and cardiomyopathy [61, 62].

### Endoplasmic reticulum stress

The endoplasmic reticulum (ER) has a significant role in protein, lipid, glycogen, and calcium metabolism [63]. Lipotoxicity, through increasing reactive oxygen species and oxidative stress, can trigger ER stress and, consequently, the accumulation of unfolded proteins in ER [64]. Normally, unfolded protein response (UPR) compensates for this stress, and the ER restores its normal function in maintaining protein and lipid homeostasis [65]. Nevertheless, in the case of prolonged stress, as in lipotoxicity, ER stress can lead to apoptosis [66]. ER stress can also induce insulin resistance [67] and anabolic intolerance in skeletal myocytes [68].

## HISTOLOGICAL CRITERIA OF LIPOTOXICITY-RELATED SARCOPENIA

Quantitative histological staining using Sudan black dye reveals myosteatosis as one of the most apparent histological changes occurring in lipotoxicity-related sarcopenia. The relative fat mass in muscles can be measured using the Lipid Accumulation Index (LAI) and quantified as described previously [55]: total area with lipid droplets of muscle fiber × 100/total cross-sectional area of muscle fiber. Myofiber atrophy has been histologically defined in obese rat skeletal muscles as heterogenicity in the cross-sectional areas of the myofibers [57]. In elderly humans with sarcopenia, the fast-twitch myofibers show considerable reductions in diameter [69].

Satellite cells decline in number and function in aging skeletal muscles [70–72]. Proteomic analysis, quantitative immunofluorescence, and ultrastructural morphological and morphometrical analysis have shown that matrisome changes accompany the aging of skeletal muscle in the form of increases in some proteins, such as collagens IV and VI and laminin. In aged rats, these changes present as more linear and larger collagen bundles in the perimysium and thickening of the endomysium of the gastrocnemius [49].

## CONCLUSION

The important repercussions of lipotoxicity in patients with sarcopenia are that it increases disability, morbidity, and mortality. Therefore, appropriate lipotoxicity management should be considered a primary target for the prevention and/or treatment of chronic musculoskeletal and other aging-related disorders. Further research advances are needed to better understand the molecular details underlying lipotoxicity and its consequences for sarcopenia and sarcopenia-related comorbidities.
